# Identifying Effective Psychotherapies for Older Adults in an Inpatient Setting: A Narrative Review and Synthesis

**DOI:** 10.1192/bjo.2024.136

**Published:** 2024-08-01

**Authors:** Inês Sequeira, Stephen De Souza

**Affiliations:** 1 Bristol Medical School, Bristol, United Kingdom; 2 Somerset NHS Foundation Trust, Taunton, United Kingdom

## Abstract

**Aims:**

We sought to review the evidence available to answer the question: Which psychological therapies are effective in the treatment of depression in older adults in an inpatient setting?

**Methods:**

An advanced literature search and systematic review was conducted using Web of Science and PubMed. A set of keywords were identified around depression, older age and the inpatient setting. These were combined with a wide range of keywords around psychological therapies.

Non-English language articles were translated using Google translate.

Articles were reviewed for the relevance to the study question by reviewing the title and abstract. Full text articles were retrieved for those felt to be relevant to the study question.

**Results:**

Of 709 articles identified from both databases, 20 articles were retrieved for full text review. Five studies were identified that appeared to offer insight into the study question. These papers focused on interpersonal therapy, cognitive behavioural therapy, or behavioural group therapy.

Brand and Clingempeel (1992) investigated the incremental implementation of group behavioural therapy in a randomized control trial. The study did not show statistically significant differences between treatment groups, but clinical significance differences supported this intervention's efficacy.

A case study by Soller (1997) followed the journey of a 69-year-old man through inpatient CBT sessions over three and a half months. This was followed with outpatient follow up. There was improvement but this was primarily subjective reporting.

A randomized controlled trial by Snarski et al. (2011) looked at the efficacy of behavioural therapy. The authors' overall conclusion was that patients benefit from this intervention and that further investigations should be done to strengthen their findings further.

A pilot study by Cabanel et al. (2017) focused on determining the feasibility of a multi-professional adaptation of group behavioural therapy sessions. This paper provides a signal towards the effectiveness of multi-professional approach to treatment.

Bollmann et al. (2020) focused on the implementation of interpersonal skills groups. It showed good feasibility as well as good patient adherence. Self-reported and observer-reported depression ratings saw improvement throughout the study.

**Conclusion:**

Although the studies showed a signal towards improvement for a range of therapies, the evidence from these studies is not convincing.

There is a lack of high quality research in this area. More studies are needed to determine the most appropriate psychological therapy to use and how this might be adapted to the transient nature of the inpatient setting.

